# Long‐term results of anterior cruciate ligament reconstruction using the medial third of the patellar tendon

**DOI:** 10.1002/jeo2.70483

**Published:** 2025-10-28

**Authors:** Jim Georgoulis, Olga Savvidou, Nikolaos Paschos, Paraskevi Kosta, Kostas Patras, Maria Argyropoulou, Panayiotis Papagelopoulos, Anastasios Georgoulis

**Affiliations:** ^1^ 1st Department of Orthopaedic Surgery National and Kapodistrian University of Athens Athens Greece; ^2^ Harvard Medical School Boston Massachusetts USA; ^3^ Department of Radiology University Hospital of Ioannina Ioannina Greece; ^4^ Orthopaedic Sports Medicine Center of Ioannina University of Ioannina Ioannina Greece

**Keywords:** anterior cruciate ligament, medial third graft, patellar tendon, sports participation

## Abstract

**Purpose:**

To report long‐term outcome of anterior cruciate ligament reconstruction (ACLR) using the medial third of the patellar tendon in terms of knee function evaluated with clinical assessments, patient‐reported outcomes (PROs) and radiological examinations.

**Methods:**

Forty patients were retrospectively evaluated 16.8 ± 0.3 (range 16.2–17.4) years after the index operation for anterior knee pain, side‐to‐side difference in anterior tibial translation (ATT), Lysholm score, Tegner score, subjective International Knee Documentation Committee (IKDC) score, Knee injury and Osteoarthritis Outcome Score (KOOS) and radiographic evidence of osteoarthritis (Kellgren–Lawrence osteoarthritis [OA] grading).

**Results:**

Incidence of donor‐site knee pain was 7.5% and the severity of the pain ranged 0.5–5.5 on visual analogue scale. Incidence of kneeling pain was 17.5% and the severity of pain ranged 3–7. There were no evidence for side‐to‐side differences in ATT (operated: 2.0 ± 0.7 vs. intact: 2.0 ± 0.9, *p* = 0.867). Lysholm had very large correlation with KOOS_QoL_ (0.77 [0.58; 0.90], *p* < 0.001). IKDC had very large correlations with KOOS_sports_ and KOOS_QoL_ (0.75 [0.56; 0.87], *p* < 0.001 and 0.70 [0.48; 0.85], *p* < 0.001). KOOS_sports_ and KOOS_ADL_ had the strongest correlation (0.79 [0.60; 0.91], *p* < 0.001). Approximately 55% of the sample had IKDC Z‐score higher than age‐matched population average and approximately 75%–80% of the sample was above the established PASS thresholds. The operated side exhibited more pronounced progression of knee osteoarthritis (higher K‐L grade) (*χ* ^2^ = 12.9, *p* = 0.002); however only five knees in total (four operated, one intact) had Grade 2 radiographic OA.

**Conclusions:**

Long‐term clinical outcomes following ACLR with the medial third of the patellar tendon demonstrate a low incidence of anterior knee pain, excellent anterior‐posterior stability and low rate of mild radiographic osteoarthritis. The majority of patients had achieved acceptable knee function and symptom resolution. These findings suggest that the medial third of the patellar tendon autograft is a reliable option for achieving sustained knee function and high patient satisfaction in the long term.

**Level of Evidence:**

Level IV.

**Abbreviations**
ACL: anterior cruciate ligament
ACLR: anterior cruciate ligament reconstruction
IKDC: International Documentation Knee Documentation Committee
KOOS: Knee injury and Osteoarthritis Outcome Score
OA: osteoarthritis
PASS: patient acceptable symptom state
PRO: patient reported outcome
QoL: quality of life

## INTRODUCTION

Anterior cruciate ligament (ACL) injuries are common, particularly among young athletic populations. Surgical reconstruction of the ACL (ACLR) is considered the gold‐standard treatment for most of these patients, especially when the goal is to return to high‐demand athletic activity [28]. The purpose of the ACLR is to restore anteroposterior and rotatory stability [29] and, compared to the ACL deficient knee, to improve the biomechanical load‐bearing of the knee joint under stress (e.g., athletic manoeuvres) [28].

The most commonly used grafts are the patellar tendon, harvested either as the central third [14] or the medial third [12, 22, 24], the hamstrings tendons, used as a four‐strand graft or as a quadrupled semitendinosus graft [5, 6] and, in recent years, the quadriceps tendon graft [3]. Although ACLR graft selection is a multifactorial decision influenced by several factors, such as donor‐site morbidity, functional deficits, patient‐reported outcomes, re‐rupture and infection rates [5, 16, 26, 27], the bone‐patellar‐tendon‐bone (BPTB) graft is well researched and offers several advantages [6, 41, 43, 44].

The long‐term outcomes following ACLR with a BPTB autograft have been well described in the literature; [9, 11, 34, 40, 42] good objective clinical results with normal or near normal knee stability and satisfactory subjective patient reported outcomes (PROs) for the majority of patients. Moreover the rates of revisions and clinical failures are low (respectively, ~8% and ~5%), whereas the presence of signs of osteoarthritis (OA) is high with a 2.8 RR for operated leg compared to the healthy knee [13].

The available data on long‐term outcomes following ACLR with BPTB have primarily evaluated the middle (central) third of patellar tendon [9, 11, 40, 42], whereas long‐term outcomes using the medial third are very limited [35]. Notably, the index operation in the above study on the medial third was performed circa 1970; since then, more advanced ACLR techniques have been introduced [12, 22]. A recent study suggested that, in addition to graft‐type, graft‐harvesting techniques may also affect donor‐side morbidity and patient‐reported outcomes [14]. Furthermore, potential advantages of using the medial third of the patellar tendon, compared to the middle third of the patellar tendon, include the use of a single incision on the patellar tendon and a lower risk of patellar lateral maltracking, patellar tendon rupture or shortening, and patellar fracture [22, 24].

Therefore, the aim of the present study was to evaluate the long‐term outcomes of knees reconstructed with an ACL graft using the medial third of the patellar tendon. Our research hypothesis was that the medial‐third of the patellar tendon autograft would be associated with favourable clinical and patient‐reported outcomes in the long term.

## MATERIALS AND METHODS

### Patients

This study was a retrospective case series involving clinical, PRO and radiological assessments. Inclusion criteria were i) skeletally mature patients at the time of injury (aged 18‐40 years) and ii) pre‐injury participation in Level I or II sports at least twice per week. Prior to surgery, a complete unilateral ACL injury and any concomitant injuries were verified with magnetic resonance imaging. Patients with more than 25% meniscal damage were excluded. Patients with multi‐ligament injuries, revision ACLR, or severe chondral lesions (Outerbridge grade III or IV) were also excluded, as the severity of these concomitant injuries could negatively affect outcomes. Written informed consent was obtained from all patients, and approval was granted by the regional institutional review board.

### Surgical procedure

**Figure 1:**
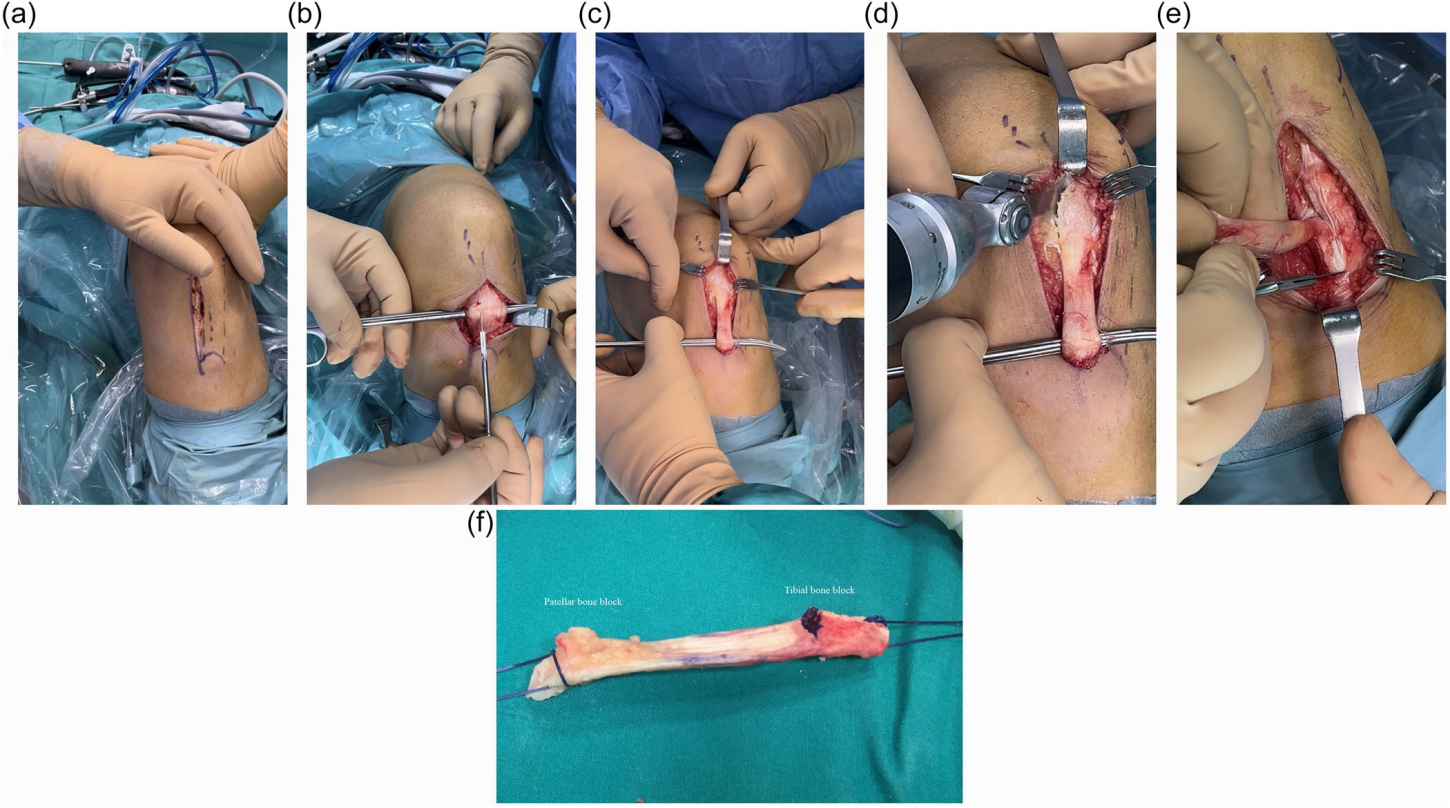
Harvesting site of the medial third of the patellar tendon (left knee). (a) Vertical medial incision; (b) tendon exposure; (c) tendon preparation; (d) preparation of the patellar bone block; (e) preparation of the tibial bone block; (f) medial third of the patellar tendon and the accompanying bone blocks.

The graft was harvested from the medial third of the patellar tendon [12]. A vertical medial incision approximately 6–8 cm in length was made, extending from the inferior‐medial edge of the patella to the medial aspect of the tibial tuberosity (Figure 1a). After dissection of the subcutaneous tissues, the patellar tendon was fully exposed (Figure 1b,c). The autograft included the medial third of the patellar tendon together with two bone blocks: one from the inferior pole of the patella (10 × 10 mm) and one from the tibial tuberosity, approximately (20 × 25 mm in size) (Figure 1d–f). The graft was harvested using an osteotome and precision saw aiming for a 10 mm diameter. Particular care taken to avoid fractures, especially of the patella. Due to the small size, the patellar defect was not grafted; whereas the tibial defect was grafted. Graft preparation involved shaping the bone blocks to fit precisely into the bone tunnels subsequently created in the femur and tibia. Two holes were drilled in each bone block to allow passage of the sutures. The graft was stored in saline with vancomycin until implantation. The femoral bone tunnel was created arthroscopically through the anteromedial portal with the knee flexed to 120° flexion, and positioned at the center of the anatomical ACL insertion. The tibial bone tunnel was positioned 2 mm posterior to the center of the native ACL insertion. The graft was placed in the femoral tunnel with the cortical surface of the tibial bone block facing the roof of the intercondylar notch (over‐the‐top position). Fixation was achieved with bioabsorbable screws in both the femur and tibia, ensuring appropriate tension and anatomical alignment of the graft. Final tibial fixation was performed with the knee at 30° flexion while maintaining graft tension. A final assessment through the full range of motion confirmed the absence impingement against the intercondylar roof, lateral femoral condyle, or posterior cruciate ligament [12].

### Post‐surgical treatment

All ACLR patients followed the same standardised rehabilitation protocol. Isometric training of the thigh muscles was initiated on the first postoperative day. Knee joint was permitted from 0° to 90° of flexion during the first 2 weeks. Patients were mobilised with crutches and an extension brace for 4 weeks. Step‐wise weight bearing began after postoperative Week 4, with full weight bearing allowed by Week 6. Jogging was permitted after 2 months. Before returning to sporting activities, patients underwent proprioceptive training. Return to sports was allowed at 6–9 months postoperatively, provided that full functional strength and stability had been regained. Strength at that time was evaluated with an isokinetic dynamometer (BIODEX System‐3, Biodex Corp, Shirley, New York), which demonstrated acceptable quadriceps and hamstring strength symmetry. During the return‐to‐sport phase, participation in sport‐specific training was gradually increased with milestones defined as a limb symmetry index (LSI) of ≥90% for both strength and hop performance [21].

### Data collection

Clinical assessments were performed by an independent orthopaedic surgeon with extensive experience in objective knee evaluation. Assessments included the presence of donor‐site knee pain and pain on kneeling (recorded as yes/no) and pain severity measured using a 10‐cm visual analogue scale (VAS). Anterior tibial translation was also evaluated using the KT‐1000 knee arthrometer (MEDmetric Corp, San Diego, California) at 134 N.

For PRO assessment, patients completed the Tegner and Lysholm activity scores, the IKDC form (IKDC score) [1, 18] and the KOOS form (KOOS score) [31]. The IKDC form is scored on a scale of 0–100 and demonstrates good test‐retest reliability, construct validity and responsiveness in individuals following ACL injury [18, 19], with extensive normative data available [1]. The KOOS consists of five subscales: pain, other symptoms, function in daily living, function in sport and recreation (Sport/Rec), and knee‐related quality of life (QoL). All subscale scores were calculated according to published guidelines and range from 0 to 100 [31]. The KOOS demonstrates good internal consistency, test‐retest reliability, construct validity, and responsiveness in patients following after ACL injuries and reconstruction [8, 32].

All patients underwent radiographic examination of both the index and contralateral intact knee. Standard weight‐bearing anteroposterior and lateral views of the tibio‐femoral compartment were obtained. Radiographs were used to assess OA progression according to the Kellgren–Lawrence classification system (K‐L grading) [35, 42]. Definitions for each grade were as follows: Grade 0, no changes; Grade 1, doubtful narrowing of the joint space and possible osteophytic lipping; Grade 2 (mild), definite osteophytes and possible narrowing of the joint space; Grade 3 (moderate), multiple osteophytes, definite narrowing of the joint space and some sclerosis, and possible deformity of the bone ends; Grade 4 (severe), large osteophytes, marked narrowing of the joint space, severe sclerosis and definite deformity of the bone ends [35]. K‐L grades ≥ 2 were considered indicative of radiographic OA [35, 42]. All radiographs were interpreted by consensus by two senior musculoskeletal radiology consultants.

### Statistical analysis

Descriptive outcomes are reported as mean ± standard deviation. In addition, patients were classified as above or below the age‐matched population average using Z‐scores for the IKDC [1], and above or below the 'patient acceptable symptom state' (PASS) threshold for the IKDC, KOOS_sports_ and KOOS_QoL_ [25]. Z‐scores thresholds were defined as follows: < −0.2, below population average; −0.2 to 0.2, population average; and >0.2, above population average. PASS thresholds were: IKDC > 75.9, KOOS_sports_ > 75 and KOOS_QoL_ > 62.6 [25]. The Wilcoxon rank sign test was used to compare pre‐injury versus follow‐up Tegner activity scores. Spearman rank order correlations were employed to assess the strength of associations between Lysholm score, IKDC score and KOOS sub‐scales. Correlation magnitudes were interpreted as follows: 0.1–0.3, small; 0.3–0.5, moderate; 0.5–0.7, large; and >0.7, very large [7]. Cross‐tabulations with *χ* ^2^ tests were used to examine associations between radiographic OA grading and knee side (operated vs. intact). For all analyses, statistical significance was set at *p* < 0.05.

## RESULTS

**Figure 2:**
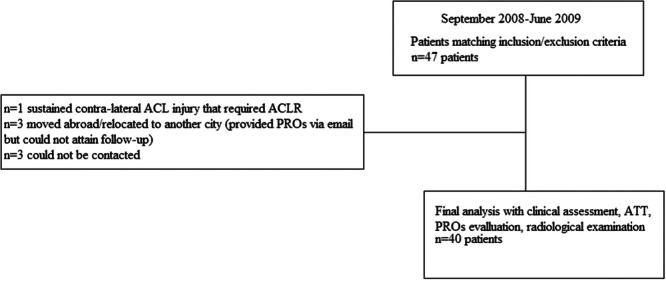
Flowchart of study patients. ACLR, anterior cruciate ligament reconstruction; ATT, anterior tibial translation; PROs, patient‐reported outcomes.

Forty seven patients who met the inclusion criteria underwent arthroscopically assisted ACLR by the same orthopaedic surgeon between September 2007 and June 2008, using the medial third of the patellar tendon. One patient sustained a contralateral ACL rupture that required arthroscopically assisted ACLR and was excluded. Three patients had relocated abroad or to another city; although they provided PROs via email, they were unable to attend the follow‐up. As their inclusion did not alter PRO results, they were excluded from the analysis. In addition, three patients could not be contacted. Thus, forty patients attended the follow‐up for clinical evaluation, anterior tibial translation measurement, PROs assessment and radiological examination (Figure 2).

The follow‐up evaluation was conducted at a mean of 16.8 ± 0.3 years after the index operation. All patients were male, with a mean age at the time of index operation of 25.0 ± 6.7 years, and a mean age of 41.6 ± 6.6 years at follow‐up. During the index operation, a small posterior meniscal flap (< 25%) was removed in four patients, and two patients underwent a partial meniscectomy (< 25%) on the operated leg. In addition, two patients had a partial meniscectomy (< 25%) on the operated leg. Patients' characteristics are summarised in Table 1.

**Table 1:** Patient demographics.

Demographics	
Age at operation, y	41.6 ± 6.6
Height, cm	179 ± 3
BMI, kg·m^−2^	25.9 ± 3.4
Injured knee, *n*	23 right/17 left
Gender, %	100 male/0 female

Abbreviation: BMI, body mass index.

The incidence of donor‐site knee pain was 7.5% (3 of 40 patients), and the severity was low (range, 0.5–5.5 out of 10). The incidence of kneeling pain was 17.5% (7 of 40 patients), with somewhat higher severity (range, 3–7 out of 10). No significant side‐to‐side differences were observed in anterior knee translation (operated: 2.0 ± 0.7 mm vs. intact: 2.0 ± 0.9 mm; *p* = 0.867). Observed mean ± SD scores for the Lysholm score, IKDC score and the KOOS sub‐scales are presented in Table 2. Patient reported Tegner activity score had a median (IRQ) of 7 (6–9) prior to injury and 5 (4–6) at follow‐up (*p* < 0.001).

**Table 2:** Descriptive statistics for the patient reported outcomes.

	*n*	Mean	SD
Tegner_score_ ^a^	40	5.0	4–6
Lysholm_score_	40	91.9	10.0
IKDC_score_	40	87.2	13.3
KOOS_symptoms_	40	91.3	7.8
KOOS_pain_	40	94.3	1.0
KOOS_ADL_	40	96.5	6.2
KOOS_sports_	40	85.8	20.1
KOOS_QoL_	40	83.7	23.2

*Note*: Data are reported as mean ± SD.

Abbreviations: IKDC, International Documentation Knee Documentation Committee; IQR, interquartile range; KOOS, Knee injury and Osteoarthritis Outcome Score; QoL, quality of life; SD, standard deviation.

^a^ For Tegner score, median and IRQ values are reported.

There were large to very large correlations between Lysholm score, IKDC score and the KOOS sub‐scales. The largest correlation was observed between KOOS_ADL_ and KOOD_sports_ (Table 3).

**Table 3:** Interrelationships between Lysholm, IKDC and KOOS sub‐scales.

	Tegner_score_	Lysholm_score_	IKDC_score_	KOOS_symptoms_	KOOS_pain_	KOOS_ADL_	KOOS_sports_	KOOS_QoL_
Tegner_score_	1.00	0.06 (−0.24; 0.35)	0.42 (0.15; 0.65)	0.08 (−0.21; 0.36)	0.31 (0.01; 0.58)	0.28 (−0.02; 0.58)	0.32 (0.01; 0.58)	0.38 (0.08; 0.64)
Lysholm_score_		1.00	0.63 (0.35; 0.81)	0.49 (0.18; 0.74)	0.56 (0.28; 0.74)	0.64 (0.38; 0.83)	0.68 (0.42; 0.85)	0.77 (0.58; 0.90)
IKDC_score_			1.00	0.38 (0.03; 0.65)	0.57 (0.30; 0.78)	0.65 (0.39; 0.83)	0.75 (0.56; 0.87)	0.70 (0.48;0.85)
KOOS_symptoms_				1.00	0.34 (0.03; 0.60)	0.55 (0.28; 0.76)	0.54 (0.25; 0.76)	0.39 (0.07; 0.67)
KOOS_pain_					1.00	0.77 (0.53; 0.91)	0.64 (0.37; 0.83)	0.64 0.37; 0.83)
KOOS_ADL_						1.00	0.79 (0.60; 0.91)	0.68 (0.45; 0.86)
KOOS_sports_							1.00	0.75 (0.52; 0.88)
KOOS_QoL_								1.00

*Note*: Values are reported for Spearman correlation coefficient (mean [95% confidence intervals]).

Abbreviations: IKDC, International Documentation Knee Documentation Committee; KOOS, Knee injury and Osteoarthritis Outcome Score; QoL, quality of life; SD, standard deviation.

**Figure 3:**
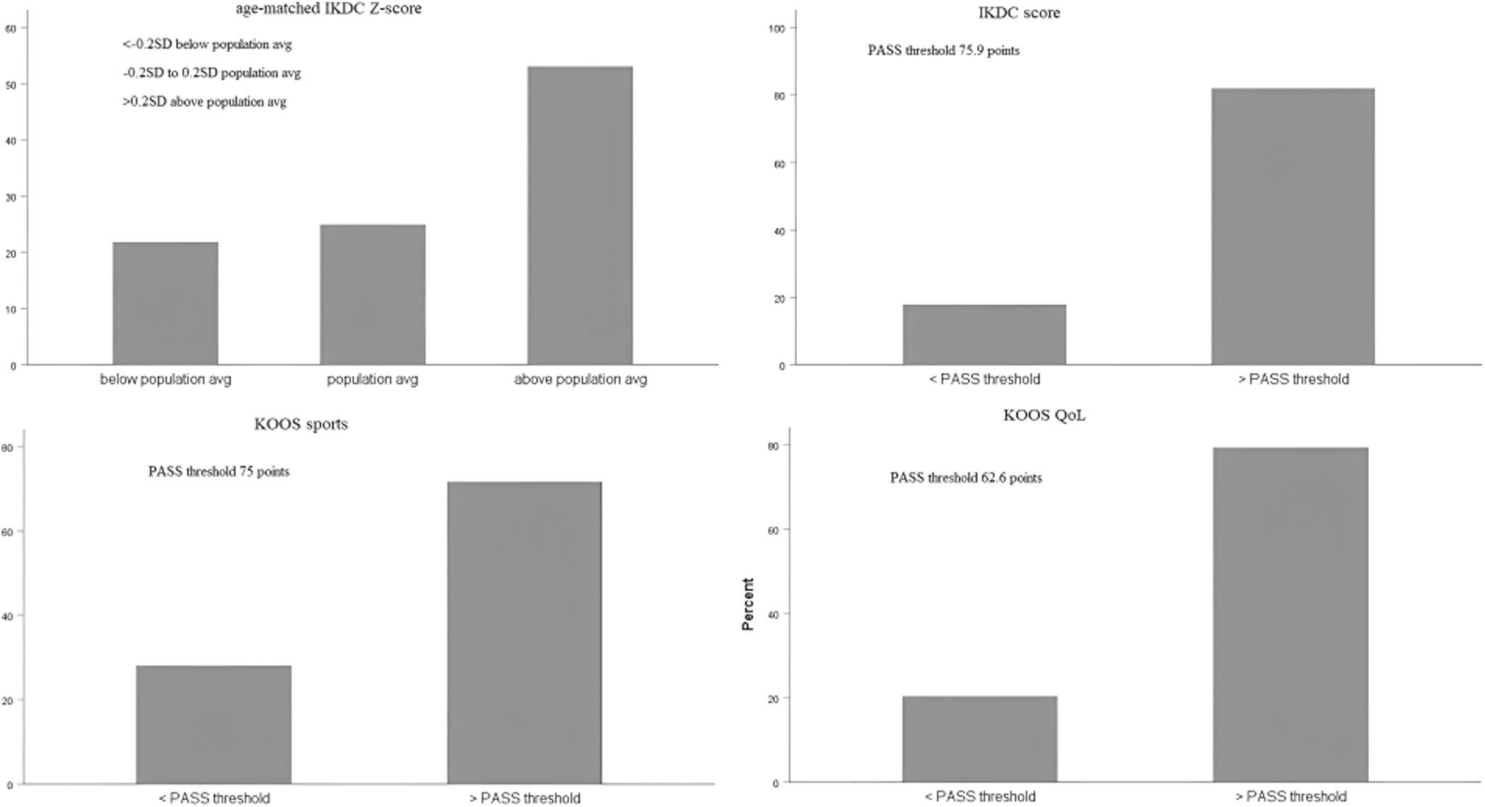
Bar graphs showing the % of patients with IKDC scores above the age‐matched population average and IKDC score, KOOSs_ports_ and KOOS_QoL_ scores above the PASS threshold. IKDC, International Documentation Knee Documentation Committee; KOOS, Knee injury and Osteoarthritis Outcome Score; PASS, patient acceptable symptom state.

According to IKDC Z‐scores, approximately 55% of the sample had IKDC Z‐score higher and approximately 20% lower than age‐adjusted population average. According to PASS criteria, approximately 75%–80% of the sample was above the established thresholds (Figure 3).

**Figure 4:**
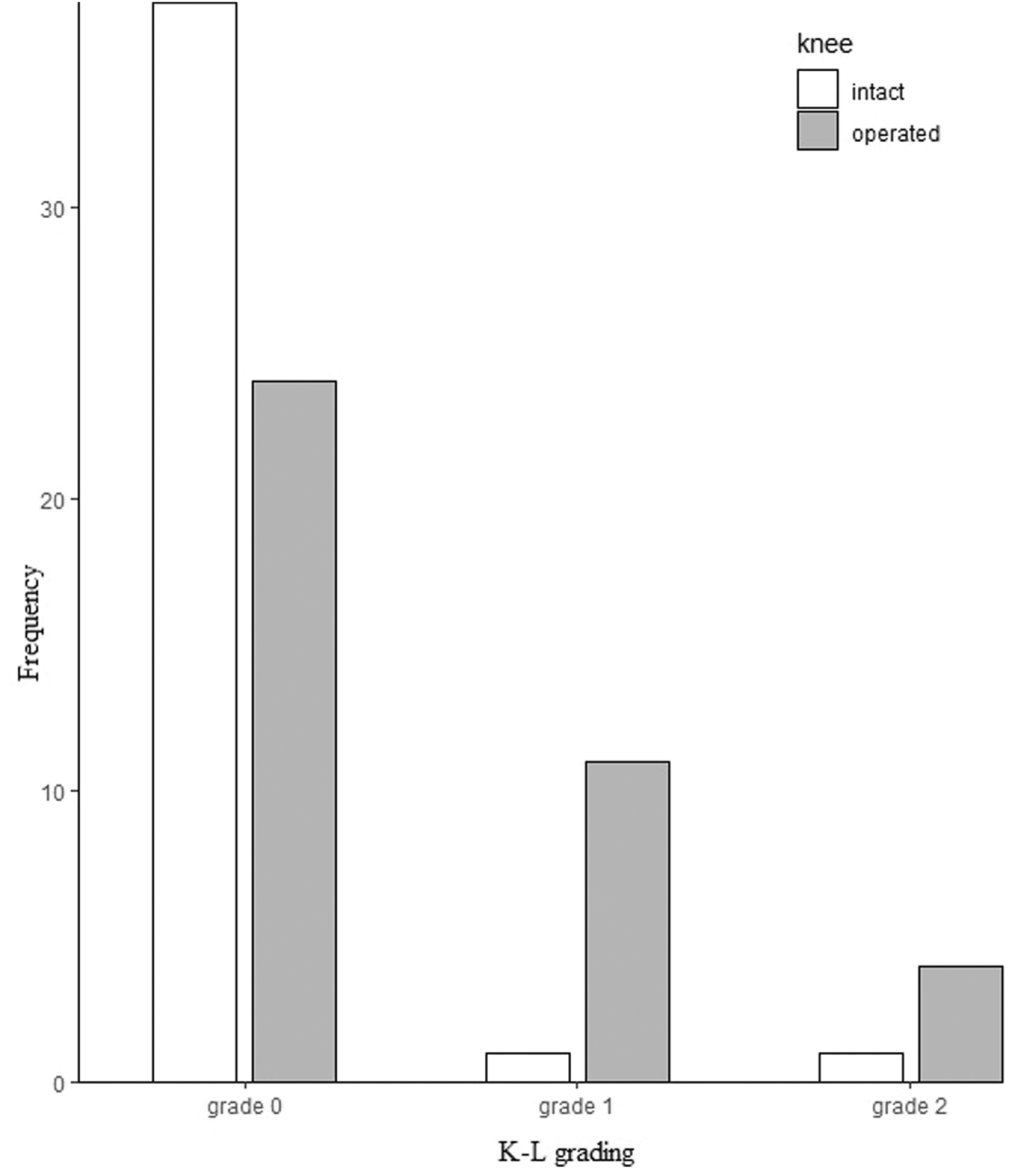
Radiographic osteoarthritis (OA) grading (Kellgren–Lawrence [K‐L]) per knee side (operated vs. intact).

The incidence of radiographic OA was 13% (5 knees in total; 4 operated, 1 intact), with all cases classified as mild (Grade 2). The operated knees demonstrated more pronounced OA progression, as indicated by higher Kellgren–Lawrence (K‐L) grades, compared with the intact knees (*χ* ^2^ = 12.9, *p* = 0.002) (Figure 4). Specifically, the proportion of Grade 0 OA was lower in the operated knees than in the intact knees (61.5% vs. 94.9%), whereas the proportion of Grade 1 OA was higher in the operated knees compared with the intact knees (28.2% vs. 2.6%).

## DISCUSSION

Our results demonstrated a low incidence of donor‐site and kneeling pain, as well as excellent anterior‐posterior stability of the operated knee, with no evidence of increased anterior tibial translation. PRO's indicated good long‐term outcomes for most patients, with 75%–80% achieving IKDC, KOOS_sports_ and KOOS_QoL_ scores above the PASS threshold [25], and nearly all patients continuing participation in some kind of sports. Although the operated knees exhibited higher K‐L grades, the severity of radiographic OA was clinically mild. Large to very large correlation among PROs further strengthening the internal validity of the results.

A key concern following ACLR is the healing of the donor‐side defect, which, in the case of BPTB grafts, has been implicated in the development of anterior knee pain [14]. Anterior knee pain, donor‐site pain and kneeling pain have been extensively studied [5, 6]. Although some authors have suggested that this effect diminishes over time [38], the generally lower incidence of such complication after hamstrings tendon ACLR is considered a primary advantage over BPTB ACLR [16, 41]. Nevertheless, studies report a wide range of postoperative donor‐site morbidity associated with BPTB grafts, with anterior knee pain rates varying from 8% to 50% [33, 38, 41, 43]. For example, Mastrokalos et al. [23] reported that approximately 60% of contralateral donor knees exhibited localised tenderness of varying degrees and proposed that the pain originated from the BPTB graft harvest rather than the ACLR itself. In contrast, a more recent study, utilising updated graft‐harvesting procedures reported a substantially lower incidence of donor‐site knee pain (13.9%) and very low rates of kneeling pain (3.7% inability to kneel on hard surfaces) [14]. Additionally, the healing process of the donor‐site defect following medial ACLR has been previously investigated in early animal models [4, 20], and Moebius et al. [24], concluded that alterations of the extensor apparatus after medial third ACLR were similar to those after middle third ACLR. Medial third ACLR avoids direct incision over the tibial tubercle and may be associated with lesser direct incisional pressure with activities such as kneeling. These potential advantages of the medial third BPTB graft are further supported by the low rates of donor‐site knee pain observed in the present study.

Regarding anterior‐tibial translation, most [2, 33, 38]—but not all [17]—report slightly lower side‐to‐side difference for BPTB autografts compared with hamstrings autografts. For example, Webster et al. [38] reported side‐to‐side differences as low as 0.6 ± 1.5 mm for the middle third of the patellar tendon at a follow‐up of 14–17 years. Our results suggest that the medial third of the patellar tendon can provide excellent long‐term anterior‐posterior knee stability, potentially even better to the middle third of the patellar tendon autograft [9, 11, 34]. It should be noted, however, that although anterior‐posterior static stability is a useful indicator of graft integrity, attempts to minimise ATT at the expense of achieving full post‐operative range of motion may in fact contribute to increased OA changes in the knee [34].

In recent years, there has been growing interest in PROs as means of quantifying the results of ACLR in the clinical setting. The IKDC, Lysholm score, Tegner scale and KOOS scales are the most frequently used instruments for PRO assessment [5, 6, 41, 43, 44]. The IKDC form is widely accepted as the standard tool for evaluating ACLR, and most available data on donor‐site morbidity are derived from this instrument. Our reported mean Tegner activity score is very similar to the 4–7 range reported in case series using the middle third of the patellar tendon at follow‐up periods of 15–20 years [9, 11, 40, 42]. It should be noted that patients' activity scores are lower than their preoperative levels; this decrease does not necessarily reflect diminished knee function, but may be also influenced by aging and associated lifestyle changes between the time of injury and follow‐up [9, 11, 40, 42]. Similarly, our reported mean Lysholm scores are comparable to those of Costa‐Paz et al. [9] and slightly higher than the 86–87 range reported by others [11, 40, 42]. Webster et al. [38] reported IKDC scores nearly identical to our case series ( ~ 87) and slightly higher than those reported elsewhere [9, 11, 40]. Our cohort also demonstrated markedly higher KOOS QoL subscales than those reported by van Yperen et al. [40], and values more similar to Costa‐Paz et al. [9]. All other KOOS subscales were comparable [9, 40]. Although the IKDC score has been reported to be superior to KOOS in capturing patients' symptoms after ACLR [15], it does not specify whether the underlying functional activities evaluated are related to donor‐site pain, joint instability, or other factors. In the present study, the IKDC showed moderate to very large correlations (*r* = 0.38–0.75) with the various KOOS subscales suggesting that the shared variance is not uniform across all subscales. In contrast, the KOOS_symptoms_ subscale demonstrated the weakest correlations (0.34–0.55) with the Lysholm, the IKDC score and, the other KOOS subscales. Although several very large correlations between the PRO′s were observed, similar published results are lacking ‐with the exception of a very large correlation between Lysholm and IKDC scores [13]. Importantly meta‐analyses typically analyse PRO′s separately assuming a priori that PRO′s within a single study are uncorrelated [13]. Future meta‐analyses should consider modelling these associations when multiple PRO′s are reported within the same study.

While the reported PRO's in their original scale (i.e., 0–100) are consistent with previous literature, they are often difficult to interpret clinically in terms of patient satisfaction with the overall outcome of the index operation [10, 37, 39]. For this reason, the concept of “patient acceptable state symptom” (PASS) was developed [25, 36]. Although the proposed thresholds for PASS do not fully agree across all PRO's, our results indicated that 75%–80% of the sample achieved an “acceptable symptom state”. This finding further supports the observation that the significant decline in Tegner activity scores does not align with the generally favourable PROs (correlations between Tegner activity score and all other PROs were ≤0.4). However, approximately 17 years after the index procedure, activity level is difficult to interpret, as a natural decline in athletic participation is expected due to factors unrelated to knee function, such as aging and social circumstances. Thus, whilst Tegner provides a single number, the KOOS_sports_ subscale may be more appropriate for assessing the broader dimension of physical activity and sports participation. This is further supported by the large correlations observed between KOOS_sports_ and both KOOS_ADL_ and KOOS_sQoL_.

A recent meta‐analysis reported a high rate of knee OA 20 years after ACLR (73%), with a relative risk of 2.8 compared with the contra‐lateral knee; however, only 13% of patients had severe OA [13]. Previous studies using the middle third of the patellar tendon have reported highly variable long‐term prevalence of radiological [9, 11, 40]. Thus, it has been suggested that ACLR may not prevent the long‐term development of knee OA, and several predictors have been associated with increased risk of OA progression [34]. Some authors have suggested that the nearly threefold increased risk of developing OA in the operated knee compared with the uninjured knee within 20 years since the index operation should be communicated to patients [13]. In our study, with a slightly shorter follow‐up period, there were no evidence of severe radiographic OA, and the prevalence of mild radiographic OA did not differ between operated and intact leg. However, the operated leg was less likely to appear completely normal. It should also be noted that a significant increase in radiographic OA between the 15‐ and 20‐year follow‐up periods has been reported in up to 22% of patients [30]. Shelbourne et al. [34] identified several risk factors for long‐term knee OA, with lack of full extension demonstrating the highest odds ratio (OR 3.84 for OA presence and OR, 3.86 for moderate to severe OA). Finally, although we did not observe any re‐ruptures, this could be attributed to reduced sports participation and, consequently, lower risk exposure. Similarly, Risberg et al. [30] reported only one ACL re‐rupture among 168 patients between the 15‐ and 20‐year follow‐up evaluations.

There are several limitations to this study that should be acknowledged. The retrospective, non‐randomised design and the absence of a control group may have introduced selection bias. The relatively small sample size limits the generalisability of the findings; however, this was partly due to the strict inclusion criteria applied to ensure analysis of a well‐defined patient group. Finally, the radiographic OA assessment was restricted to the tibio‐femoral compartment and potential changes in the patellofemoral compartment were not evaluated.

## CONCLUSION

Long‐term clinical outcomes following ACLR with the medial third of the patellar tendon demonstrate a low incidence of anterior knee pain, excellent anterior‐posterior stability and low rate of mild radiographic osteoarthritis. The majority of patients had achieved acceptable knee function and symptom resolution. These findings suggest that the medial third of the patellar tendon autograft is a reliable option for achieving sustained knee function and high patient satisfaction in the long term.

## AUTHOR CONTRIBUTIONS

Jim Georgoulis designed the search strategy, conducted the search, screened the papers, performed the statistical analysis, extracted the data and wrote the manuscript. Olga Savvidou assisted in designing the search strategy, reviewed the findengs and contributed to the discussion Nikolaos Paschos and Paraskevi Kosta assisted in conducting the search and screened the papers Kostas Patras extracted the data and assisted in the statistical analysis Maria Argyropoulou and Panayiotis Papagelopoulos reviewed the findings of the analysis and contributed to the discussion. Anastasios Georgoulis assisted in designing the search strategy, reviewed the findings of the analysis and contributed to the discussion. All authors reviewed and approved the final manuscript.

## CONFLICT OF INTEREST STATEMENT

The authors declare no conflict of interest.

## ETHICS STATEMENT

Attikon University General Hospital Scientific Board, ΑΡΘΟΠ, ΕΒΔ 4/18.01‐2021. All patients provided informed consent.

## Data Availability

The data that support the findings of this study are available from the corresponding author upon reasonable request.
